# Limitations at the Limit? Diminishing of Genetic Effects in Norway Spruce Provenance Trials

**DOI:** 10.3389/fpls.2019.00306

**Published:** 2019-03-13

**Authors:** Marcin Klisz, Allan Buras, Ute Sass-Klaassen, Radosław Puchałka, Marcin Koprowski, Joanna Ukalska

**Affiliations:** ^1^Department of Silviculture and Genetics, Forest Research Institute, Sêkocin Stary, Poland; ^2^Forest Ecology and Forest Management, Wageningen University & Research, Wageningen, Netherlands; ^3^Faculty of Biology and Environment Protection, Nicolaus Copernicus University, Toruñ, Poland; ^4^Biometry Division, Department of Econometrics and Statistics, Faculty of Applied Informatics and Mathematics, Warsaw University of Life Sciences, Warsaw, Poland

**Keywords:** radial growth, G × E, *Picea abies*, drought, phenotypic plasticity

## Abstract

Provenance trials are used to study the effects of tree origin on climate-growth relationships. Thereby, they potentially identify provenances which appear more resilient to anticipated climate change. However, when studying between provenance variability in growth behavior it becomes important to address potential effects related to site marginality in the context of provenance trials. In our study we focus on provenance-specific climate sensitivity manifested under marginal growth conditions. We hypothesized that the provenance effects are masked if trials are located at marginal environmental conditions of the natural species distribution. Under this framework, we investigate 10 Norway spruce provenances growing at two contrasting locations, i.e., a relatively drought-prone site in western Poland (at the climatic margin of Norway spruce’s natural distribution) and a mild and moist site in north-eastern Poland (within its natural range). Combining principal component analysis with climate-growth relationships, we found distinguishable growth patterns and climate correlations among provenances. That is, at the mild and moist north-eastern site, we observed provenance-specific growth patterns and thus a varying drought susceptibility. In contrast, at the dryer western site, provenance-specific growth patterns were less pronounced and all provenances expressed a common and strong sensitivity to drought. Our results indicate that the genetic specificity of growth reactions diminishes toward the distributional margins of a given species. We conclude that the climate conditions at the margins of a species’ distribution are constraining tree growth independently of tree origin. Because of this, the marginality of a site has to be considered when evaluating climate sensitivity of provenances within trials. As a consequence, the yet different responses of provenances to adverse growing conditions may synchronize under more extreme conditions in course of the anticipated climate change.

## Introduction

Predicted climate change and increased frequency of extreme weather events may result in sudden, large scale tree-dieback (Allen et al., 2010; Anderegg et al., 2012, 2015; Choat et al., 2012) rather than a gradual shift of species distribution. Sudden, large scale tree dieback is especially relevant at the margins of a species distributional range (Mátyás, 2006). This becomes particularly important if predicted rates of evolutionary response of sessile, long-living organisms (such as trees) are much slower than the predicted rate of climate change (Etterson et al., 2001). In this context, phenotypic plasticity is crucial to survive under changing environmental conditions since it results in a higher fitness of phenotypes which are better adapted to prevailing conditions (Thompson, 1991; Dudley, 2004). In recent years the frequency of drought and heat events has been increasing, however expressing a large spatial heterogeneity (Steinkamp and Hickler, 2015). Moreover, trees response to drought stress also shows great diversity among forest types, tree species, and provenances (Alberto et al., 2013; Vicente-Serrano et al., 2014). Given this variability and the associated uncertainty, forestry has to face the challenge to select tree species and provenances which are able to cope with diverse climate conditions (Bolte et al., 2009).

Although Norway spruce (*Picea abies* (L.) H. Karst) has expressed drought-induced dieback since the 1970s it yet is considered one of the key tree species for European forestry (Roberts et al., 1989). Several hypotheses indicate different biotic and abiotic environmental factors determining spruce decline (Grodzki, 2010; Hentschel et al., 2014). According to recent studies on climate adaptation of tree species in Europe, Norway spruce is supposed to be more vulnerable to climate change than other more drought-tolerant species e.g., *Abies alba* Mill. and *Pseudotsuga menziesii* (Mirb.) Franco (Lévesque et al., 2013; Zang et al., 2014; Vitali et al., 2018). Furthermore, water shortage during the growing season considerably increased Norway spruce vulnerability to bark beetle attack (*Ips typographus* L.) (Netherer et al., 2015). In addition, a recent projection of European tree-species distributions, indicates a large decline of Norway spruce abundance in Central Europe (Buras and Menzel, 2019). Thus, the risk of maladaptation to current and future climate is likely, unless forest management strategies incorporate climate-based seed transfer, thereby utilizing the phenotypically more suitable provenances (Isaac-Renton et al., 2014; Frank et al., 2017). In this context, soil properties may also significantly influence Norway spruce growth performance (Lévesque et al., 2013; Rehschuh et al., 2017).

The observed and projected negative effect of climate change on the condition and productivity of forests justifies incorporating an adaptation strategy into forest management. The most powerful tool for studying the genotype and environment interaction effect (G × E) in the context of tree adaptation are provenance trials and common garden experiments (Mátyás, 1994; Pâques, 2013). Although studying between-provenance variation on replicant experiments may provide useful simulation of environmental change over time (Mátyás, 1997) the diminishing effect of marginal environmental conditions can make it impossible to detect a provenance-specific, climate-related adaptive response. Within this context, marginal environmental conditions refer to climatic properties that represent the climatic margins of a species distributional range (Mellert et al., 2016). Differences in adaptive traits both within and among populations are observed even at the climatic margin of the species distribution (Skrøppa and Johnsen, 1999; Savolainen et al., 2004). However, under optimal growing conditions differences among provenances are pronounced, whereas under more adverse growing conditions, differences among phenotypes may diminish (Mátyás, 1994). These findings suggest a tendency toward uniform growth reactions under unfavorable (i.e., marginal) environmental conditions regardless of the genotype. That is, depending on the marginality of the trial or common garden site, phenotypic differences might be reduced (at the margins) or pronounced (in the center of the species distributional range). Consequently, site-specific environmental conditions related to climate but also soil properties and hydrology should be taken into consideration (Mátyás and Yeatman, 1992). According to Liebig’s law of the minimum (Liebig, 1840) plant productivity reflects the variation of the limiting factor (e.g., growing season temperature at high elevations and latitudes), which may cause between-provenance adaptive variation to become negligible. Moreover, if the adaptation process is modified by multiple environmental factors, the rate and the course of the process of adaptation is limited by the pace of the “slowest” factor, i.e., the factor which features the slowest rate of change in relation to other environmental factors (Blackman, 1905). This may be explained by the effect of the relatively slower factor (with a slower growth rate) to condition the influence of other factors. In this context, a complex limiting factor is drought stress as a secondary effect of different types of climate induced stress, e.g., heat waves, soil water depletion, intensive solar radiation, and their combination (Taiz and Zeiger, 2010). Consequently, when tracing phenotypic adaptation of trees to drought not only intensity but also duration of the prevailing stress factor should be considered.

Within this context, we here address a central question related to the appropriate selection of tree provenances for climate-smart forestry (Nabuurs et al., 2018):

Does species-specific marginality of a site affect provenance-specific climate sensitivity within provenance trials and if so, how?

Based on existing studies outlined above, we hypothesize that provenance-specific growth reactions will diminish toward the climatic marginality of the considered tree species. If so, this would have important implications for provenance trials in the context of adapting forestry to the anticipated climate change.

## Materials and Methods

### Study Sites and Sample Acquisition

**FIGURE 1:**
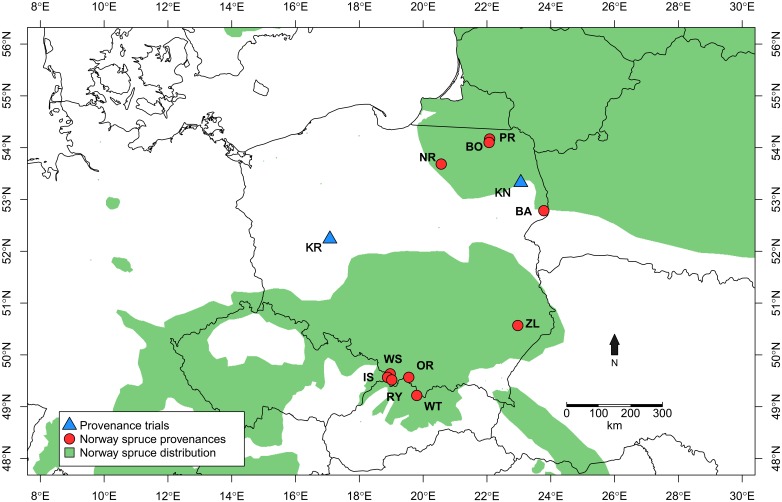
Location of study sites. Blue triangles – experimental sites, red circles – Norway spruce provenances. KR, KN – Kórnik and Knyszyn testing site; BA, BO, PR, NR, IS, WS, RY, OR, WT, and ZL Norway spruce provenances; green area – natural distribution area of Norway spruce (European Forest Genetic Resources Programme [EUFORGEN], 2009).

The study material is part of the International Union of Forest Research Organizations (IUFRO) Spruce provenance trial established in 1972 within the framework of a cooperation among research institutes from 10 European countries and Canada (Matras, 2009). 10 Polish provenances included in the provenance trial series represent the southern, northern and central range of Norway spruce in Poland (Figure 1) and the highest possible genetic variation among Polish provenances (Klisz et al., 2019). Out of altogether four provenance trials located in Poland, two were selected: Kórnik (KR; N 52.237291° E 17.076364°) and Knyszyn (KN; N 53.327119° E 23.060735°).

**FIGURE 2:**
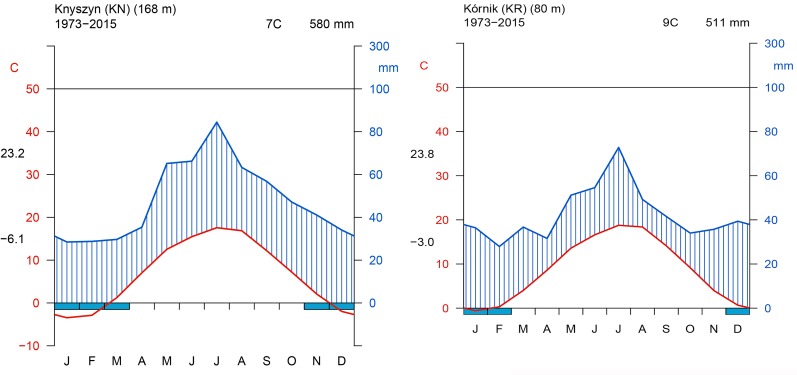
Walter and Lieth climatic diagram of study sites. Left panel – Knyszyn site, Right panel – Kórnik site.

**FIGURE 3:**
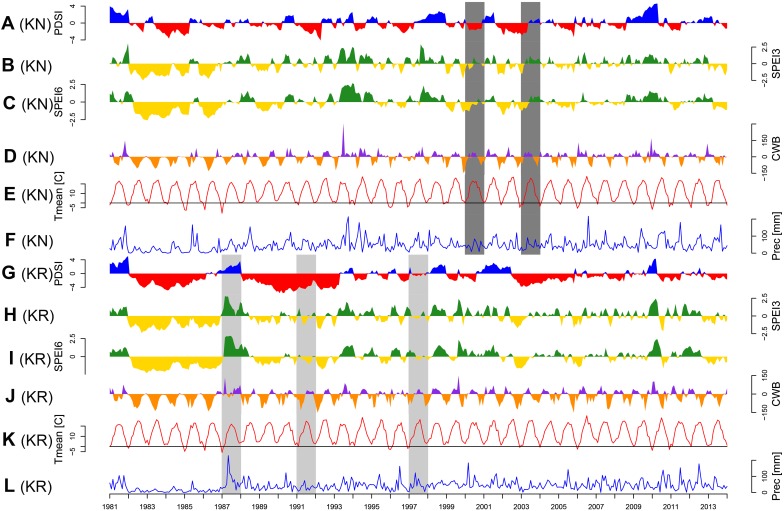
Temporal variations of main climatic parameters: Palmer Drought Severity Index (PDSI; **A** and **G**), standardized precipitation evapotranspiration index integrated over three (SPEI3; **B** and **H**) and six months period (SPEI6; **C** and **I**), climatic water balance (CWB; **D** and **J**), monthly mean temperature (Tmean; **E** and **K**) and monthly precipitation sum (Prec; **F** and **L**). Panels **A–F**: Knyszyn site, panels **F–K**: Kórnik site. Common pointer years (CPY, more than five provenances) are indicated by light gray (positive) and dark gray (negative) poligons.

The site selection aimed at representing the diversity of climatic conditions and site conditions in relation to the natural distribution area of Norway spruce. Although the average annual climate parameters and climatic water balance (CWB) of the growing season of both sites were similar, the Walter–Lieth annual aridity index (WAI) indicates that KR is a relatively drought-prone site while KN is mild and moist (29.6 and 25.7, respectively). Data from weather stations in Gorzów and Białystok, for the reference period 1973–2015 [National Oceanic and Atmospheric Administration agency (NOAA/NCEI/CWC^1^)] was used to determine the climatic conditions of provenance trials (Figure 2). Climatic conditions of KR can be considered more marginal/limiting according to the ecological requirements of Norway spruce (Tjoelker et al., 2007). To explore climate-growth relationships, total sum of monthly precipitation (P), and mean monthly temperature (T) from Gorzów and Białystok meteorological stations (years 1981–2014) were obtained from the European Climate Assessment and Dataset (ECA&D) project (Klein Tank et al., 2002) for KR and KN, respectively. Walter–Lieth annual aridity index (WAI) was calculated for each site to determine their relative water surplus or deficit. To detect periods with negative water balance, we further computed the standardized precipitation evapotranspiration index (SPEI), while the Palmer drought severity index (PDSI) was extracted from the Climate Explorer^2^. The PDSI varies slowly underlining the accumulation effect of long-term hydrological drought (Palmer, 1965). Integrated over three and six month periods, SPEI (SPEI3 and SPEI6, respectively) is defined as a standardized difference between monthly precipitation and potential evapotranspiration (PET) (Beguería and Vicente-Serrano, 2013; Beguería et al., 2014). Moreover, for each site we computed the monthly CWB for the period 1981–2014 (Figure 3). According to Thornthwaite (1948), CWB is the difference between total precipitation and PET. Due to the limited availability of meteorological data, the Hargreaves equation was used to estimate PET, which is an alternative for the FAO 56 Penman–Monteith method (Hargreaves and Samani, 1985; Allen et al., 1998):

$$PET=0.408\times 0.0023(T_{mean}+17.8){\left(T_{\max}-T_{\min}\right)}^{0.5}R_{a}$$

where *PET* is the potential evapotranspiration, *T*_mean_ is the mean of *T*_max_ and *T*_min_, *R*_a_ is extraterrestrial radiation, and 0.408 being a factor to convert units of MJ m^-2^ day^-1^ into mm day^-1^.

Regarding soil fertility, sites were relatively similar, i.e., a Mollic Gleysol in KR and a Cambisol in KN (IUSS Working Group, 2015). The provenance trials were designed according to the scheme of a random block design with four replicates, with 3-year-old seedlings with 1.65 × 1.45 m spacing. For each of the 10 provenances, 15 trees were sampled at breast height (1.3 m) for two increment cores at the end of the growing season in 2015, resulting in 150 trees from KN but only 134 from KR. The lower sample size in KR is to be explained by the fact that for three provenances (BO, NR, and RY) the number of trees suitable for sampling was lower than ten since we avoided sampling of suppressed trees, characterized by symptoms of fungal infection as well as trees located near gaps.

### Ring-Width Analyses

#### Sample Preparation

To prepare the sample material for tree-ring analysis, cores were manually surfaced, polished with abrasive paper (grain size up to 1000) and scanned (Epson Expression XL12000) at 1200 dpi resolution. Ring widths were measured with an accuracy of 0.01 mm and then cross-dated with “CooRecorder” and “CDendro” software (version 9.0, Cybis, 2017). The two series per tree were averaged into one series resulting in altogether 274 ring-width series. Individual tree-ring series were detrended using a cubic-smoothing spline with a 50% frequency cut-off at 30 years (Cook and Peters, 1981; Speer, 2010). To remove temporal autocorrelation and to emphasize the high-frequency signal (year-to-year variability) of the tree-ring series, the first-order autoregressive model (aka prewhitening) was applied to each series (Cook and Kairiukstis, 1992) finally resulting in indexed ring-width series (RWI). Using RWI, mean chronologies were computed per provenance and site using a biweight robust mean, resulting in altogether 20 provenance-chronologies.

#### Tree-Ring Data

To characterize and qualify site chronologies, Gleichläufigkeit (glk aka coefficient of coherence, Eckstein and Bauch, 1969; Buras and Wilmking, 2015); mean sensitivity (MS, indicator of general climate sensitivity of growth) and mean inter-series correlation (mean rbar, an indicator of the strength of the common signal in growth series from individual trees within a stand) were calculated on the basis of ring-width series (Douglass, 1920; Wigley et al., 1984; Cook and Kairiukstis, 1992).

#### RW Variation Between Sites and Provenances

To determine the effects of site, provenance, and year on radial growth variation, a generalized estimating equation (GEE) was used (Liang and Zeger, 1986). The following model was applied:

$$g(\mu_{ijk})=S_{i}+P_{j}+Y_{k}+S\times P_{ij}+S_{i}\times Y_{ik}+P_{j}\times Y_{jk}$$

where g(μ_ijk_) is the identity link function, μ_ijk_ is the single-tree ring-width index mean for the *ij*th site × provenance combination in the *k*th year (*k* = 1,…, 31); *S*_i_ is the effect of the *i*th site (*i* = 1,2); *P*_j_ is the effect of the *j*th provenance (*j* = 1,…,10); *S* × *P*_ij_ is the site × provenance interaction effect; *S* × *Y*_ik_ is the site × year interaction effect; *P*_j_ × *Y*_jk_ is the provenance × year interaction effect and *Y*_ik_ is the repeated measurements year effect. We used GEE since it efficiently handles data with repeated measurements as well as possible inter-correlations among data at the tree level (285 trees – clusters).

To estimate model parameters, “sandwich” (empirical) estimators were used, which are asymptotically unbiased even if the correlation structure is unknown. However, choosing a working correlation matrix that is closer to the truth improves the efficiency of estimates. We assumed compound symmetry for the data from the same tree. The analyses were done with SAS/STAT 14.3 software, and the GLIMMIX procedure was followed (Software suite developed [SAS] Institute, 2017).

#### Provenance Grouping

To group mean RWI provenance chronologies according to their similarity, a hierarchical clustering using Euclidean distance (root sum-of-squares of differences) as similarity measure and Ward (1963) clustering method with the criterion proposed by Murtagh and Legendre (2014) was applied. Four different clustering methods, single and complete linkage, the unweighted pair group method with arithmetic mean (UPGMA) and Ward’s method were tested according to their clustering structure of the dataset (Kaufman and Rousseeuw, 2008). Finally, Ward’s method was chosen, since it expressed the highest value of the agglomerative coefficient.

To investigate provenance-specific individualistic growth reaction of trees, we for each site computed a refined version of the principal component gradient analysis (PCGA, Buras et al., 2016). That is, we performed a pairwise PCGA only adding the RWI of two provenances for each of the possible provenance combinations per site. In each pairwise PCGA, Wilcoxon rank-sum test (Wilcoxon, 1945) was used to test whether the polar coordinates of RWI-loadings express a provenance-specific location shift which would indicate that the considered provenances express provenance-specific growth patterns in comparison to each other.

#### Provenance-Specific Climate Sensitivity

A pointer-year analysis was carried out using single-tree RWI to test whether the growth reactions to extreme events varied between provenances and sites (Schweingruber et al., 1990). The “Neuwirth” method, a window size of 5 years, and a series threshold of 65% were used as criteria for weak, strong, and extreme events, where the intensity classes refer to Cropper values of >1, >1.28 and >1.645, respectively (Cropper, 1979; Neuwirth et al., 2007). Years in which at least six of the ten provenances indicated a pointer year at the specific site were defined as common pointer years (CPY). Furthermore, to identify climatic drivers of growth variability, indexed provenance chronologies were correlated with previous year March through current year October temperature, precipitation, SPEI3, SPEI6, PDSI and CWB for 1981 through 2014.

#### Bioclimatic Variation Between Sites and Provenances

To characterize the climatic distance between the provenance origins and the trial sites, a principal component analysis (PCA) using 19 bioclimatic parameters, related to monthly and seasonal precipitation and temperature, was performed (Tables 1, 2). For this, climate data for the two trial sites KR and KN as well as for the 10 Norway spruce origins were extracted from BIOCLIM 1.4 (Hijmans et al., 2005) at a spatial resolution of 2.5 arcmin taken from the www.worldclim.org website. The selected variables contained data concerning bioclimatic indices calculated on the basis of monthly, seasonal (i.e., three-month periods) and annual values of precipitation and temperatures for the period 1970–2000. Tables 1 and 2 provide a simplified overview on the bioclimatic variables. For a detailed description of the bioclimatic variables, we refer to Hijmans et al. (2005). The variables with the strongest impact on the distribution of the provenances and sites along the principal components was identified on the basis of Pearson correlation coefficients. Clustering of provenances according to their locations on the PCA biplot were determined visually.

**Table 1:** Pearson correlation coefficients between climatic variables and the first three major components, eigenvalues and variation explained.

Bioclimatic variables	Abbreviation	PC 1	PC 2	PC 3
Annual Mean Temperature	bio1	-0.25	0.14	0.04
Mean Monthly Temperature Range	bio2	-0.14	0.44	0.21
Isothermality (bio2/bio7) (^∗^ 100)	bio3	0.08	0.51	-0.22
Temperature Seasonality (STD ^∗^ 100)	bio4	-0.23	-0.23	0.30
Max Temperature of Warmest Month	bio5	-0.26	0.09	0.12
Min Temperature of Coldest Month	bio6	-0.21	0.25	-0.38
Temperature Annual Range (bio5-bio6)	bio7	-0.24	-0.03	0.42
Mean Temperature of Wettest Quarter	bio8	-0.26	0.04	0.08
Mean Temperature of Driest Quarter	bio9	-0.22	-0.14	0.27
Mean Temperature of Warmest Quarter	bio10	-0.26	0.04	0.08
Mean Temperature of Coldest Quarter	bio11	-0.20	0.32	-0.24
Annual Precipitation	bio12	0.26	0.06	0.16
Precipitation of Wettest Month	bio13	0.25	0.14	0.18
Precipitation of Driest Month	bio14	0.26	0.05	0.04
Precipitation Seasonality (CV)	bio15	0.00	0.47	0.43
Precipitation of Wettest Quarter	bio16	0.25	0.12	0.21
Precipitation of Driest Quarter	bio17	0.26	0.02	0.04
Precipitation of Warmest Quarter	bio18	0.25	0.12	0.21
Precipitation of Coldest Quarter	bio19	0.26	-0.01	0.05
Eigenvalue		14.32	3.16	0.82
Variance explained		75.35	16.63	4.34


**Table 2:** Mathematical definition of bioclimatic indices.

Bioclimatic variables	Definition
Annual Mean Temperature	The annual mean temperature
Mean Monthly Temperature Range	The mean of the monthly temperature ranges (monthly maximum minus monthly minimum).
Isothermality (bio2/bio7) (^∗^ 100)	The ratio of the mean diurnal range (bio 2) to the annual temperature range (bio 7), multiplying by 100.
Temperature Seasonality (STD ^∗^ 100)	The standard deviation of the 12 mean monthly temperature values, multiplying by 100.
Max Temperature of Warmest Month	The maximum monthly temperature occurrence over a given year.
Min Temperature of Coldest Month	The minimum monthly temperature occurrence over a given year.
Temperature Annual Range (bio5-bio6)	Temperature variation over a given year, difference between maximum and minimum monthly temperature.
Mean Temperature of Wettest Quarter	The mean temperatures during the wettest three months of the year.
Mean Temperature of Driest Quarter	The mean temperatures during the driest three months of the year.
Mean Temperature of Warmest Quarter	The mean temperatures during the warmest three months of the year.
Mean Temperature of Coldest Quarter	The mean temperatures during the coldest three months of the year.
Annual Precipitation	The sum of all total monthly precipitation values.
Precipitation of Wettest Month	The total precipitation of the wettest month.
Precipitation of Driest Month	The total precipitation of the driest month.
Precipitation Seasonality (CV)	The ratio of the standard deviation of the monthly total precipitation to the mean monthly total precipitation expressed as a percentage.
Precipitation of Wettest Quarter	The total precipitation during the wettest three months of the year.
Precipitation of Driest Quarter	The total precipitation during the driest three months of the year.
Precipitation of Warmest Quarter	The total precipitation during the warmest three months of the year.
Precipitation of Coldest Quarter	The total precipitation during the coldest three months of the year.


All analyses were computed in “R” (R Core Team, 2015). Extraction of climatic data values for spruce sites was done with biovars function in the R package “dismo” 1.1-4 (Hijmans et al., 2017). PCA analyses as well as the biplot were created with fviz_pca_biplot functions from the “FactoMineR” 1.41 package (Husson et al., 2015). Detrending, chronology building, and calculation of chronology statistics were performed using the “dplR” package 1.6.4 (Bunn, 2008). Clustering analyses were calculated using “cluster” 2.0.7-1 package (Rousseeuw et al., 2018). Pointer-year analysis was done by the “pointRes” 1.1.3 package (van der Maaten-Theunissen et al., 2015). Integrated over three and six month periods, SPEI were calculated using “SPEI” 1.7 package (Beguería and Vicente-Serrano, 2013).

## Results

### Characteristics of RW Chronologies

**FIGURE 4:**
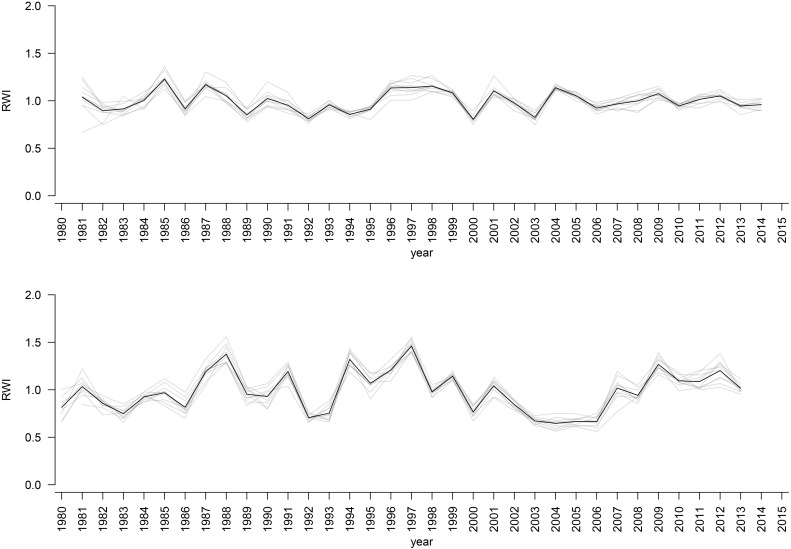
RWI provenance chronologies for KN site (upper plot) and KR site (lower plot). Black bold line: RWI mean site chronologies.

The mean sensitivity, and mean inter-series correlation varied between provenance chronologies, however, they generally featured higher values for the chronologies from KR than from the KN site (Table 3). In KR, a strong growth depression between 2003 and 2006 highlighted a bark beetle (*I. typographus L.*) outbreak (Figure 4). The results of GEE analysis (Eq. 2) for RWI confirmed the significant effect of site, provenance, and year (*P* = 0.001, *P* < 0.001, *P* < 0.001, respectively). Moreover, the interaction effects between site and provenance, site and year, as well as provenance and year were significant (Table 4; in all cases *P* ≤ 0.001).

**Table 3:** Chronology statistics of Norway spruce provenances.

Site	Provenance ID	*N*	TRW	glk	MS	*r*_bt_
KN	BA	15	3.30	0.74	0.62	0.537
	PR	15	2.98	0.75	0.72	0.560
	BO	15	2.97	0.74	0.70	0.564
	NR	15	3.33	0.71	0.61	0.452
	WS	15	3.50	0.72	0.59	0.490
	IS	15	3.46	0.74	0.54	0.417
	RY	15	2.84	0.70	0.62	0.414
	OR	15	2.89	0.73	0.58	0.500
	WT	15	2.93	0.73	0.67	0.486
	ZL	15	3.43	0.72	0.55	0.484
KR	BA	15	2.57	0.79	0.78	0.759
	PR	15	2.73	0.77	0.69	0.605
	BO	8	4.14	0.71	0.67	0.620
	NR	11	2.66	0.77	0.82	0.759
	WS	15	3.61	0.75	0.82	0.748
	IS	15	3.37	0.76	0.75	0.609
	RY	10	2.78	0.77	0.77	0.718
	OR	15	2.98	0.78	0.81	0.761
	WT	15	2.55	0.81	0.80	0.797
	ZL	15	3.40	0.72	0.76	0.684


**N* – number of trees, TRW – mean tree-ring width, glk – Gleichläufigkei, MS – mean sensitivity, *r*_bt_ – mean inter-series correlation.*

**Table 4:** Results of the GEE analysis to test for differences in ring width due to sites, provenances, years and interactions analyzed with *F* test for type 3 analysis.

Source of variation	Num Df	Den Df	*F*	*P*
Site (*S*)	1	265	11.68	0.001
Prov (*P*)	9	265	80.38	<0.001
Year (Y)	30	8090	147.42	<0.001
Site × Prov (S × P)	9	265	48.27	<0.001
Site × Year (*S* × *Y*)	30	8090	62.56	<0.001
Prov × Year (*P* × *Y*)	270	8090	1.83	<0.001


*Num Df – nominator degrees of freedom; Den Df – denominator degrees of freedom; *F* – test statistic; Site – experimental site effect; Prov – provenace effect; Year – year effect.*

### Provenance Grouping

**FIGURE 5:**
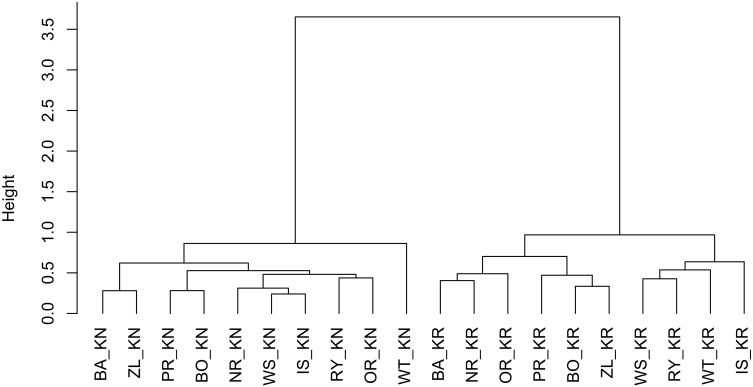
Hierarchical clustering of the RWI provenance mean chronologies, using the Euclidean distance and Ward’s minimum variance clustering method. The letters KN and KR refer to Knyszyn and Kórnik site, letters BA, BO, PR, NR, IS, WS, RY, OR, WT, and ZL refers to Norway spruce provenances.

**FIGURE 6:**
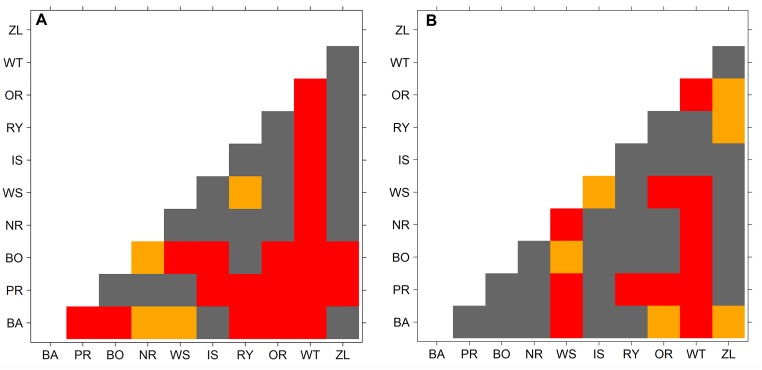
Probability value of Wilcoxon rank-sum test used to demarcate a significant difference in PCGA-ranks between provenances. Colors represent significance level of Wilcoxon rank-sum test (orange: *p* < 0.05, red *p* < 0.01, gray non-significant). Panel **A** – Knyszyn (KN) site, panel **B** – Kórnik (KR) site.

The results of the cluster analysis confirmed grouping of provenance chronologies mainly by site. However, the provenance grouping patterns within the sites were different (Figure 5). That is, at KN provenance WT took a special position relative to the remaining provenances. Moreover, the eastern provenances BA and ZL took a distinct position within the largest sub-cluster. In contrast, at KR four of the five southern provenances (WS, RY, WT, and IS) indicated a separate cluster (Figure 5). The pairwise PCGA distinguished pairs of provenances differing in terms of high-frequency growth patterns. That is, at KR the provenances WS and WT were highlighted as significantly different from the other provenances (Figure 6A and Supplementary Figure S1). The outstanding position of the provenance WT was also present at KN, although here the other, clearly distinguishable provenance was BA (Figure 6B and Supplementary Figure S2). In general, site KN expressed a higher diversity among provenances according to pairwise PCGA in comparison to KR. In other words, the differentiation between provenances was much stronger in KN compared to KR, where provenances featured more similar growth patterns.

### Climate-Growth Relationships

**FIGURE 7:**
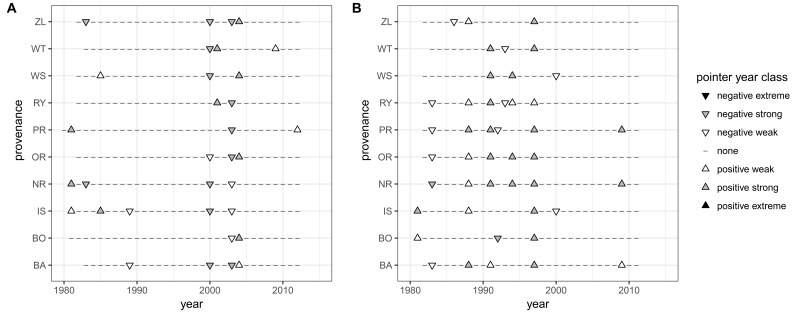
Common pointer years (CPY) for provenances, indicated by upper (positive) and inverted (negative) triangles. Panel **A** – Knyszyn (KN) site, panel **B** – Kórnik (KR) site.

**FIGURE 8:**
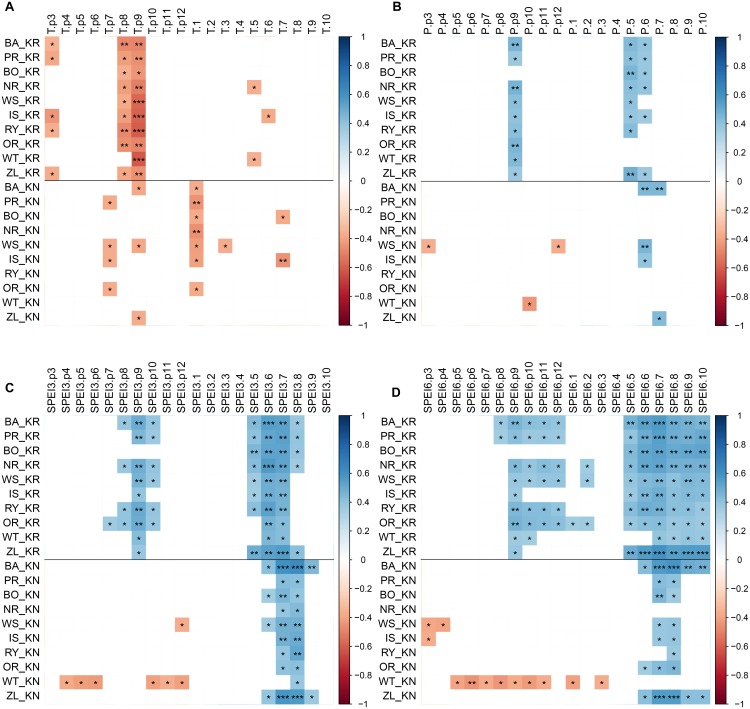
Correlations of the RWI provenance chronologies with monthly climatic parameters, previous year March through current year October: temperature (panel **A**), precipitation (panel **B**), SPEI3 (panel **C**), SPEI6 (panel **D**) over the period 1981–2014 at the two sites: KR, KN. Colors represent correlation coefficient, non-significant correlations are not represented (white), ^∗^, ^∗∗^ and ^∗∗∗^ demarcate a significance level of correlation (*p* < 0.05, *p* < 0.01 and *p* < 0.001, respectively).

The CPY clearly separated the two sites from each other since there was no similar CPY for both sites. Interestingly, the two CPY at the KN site were negative (2000 and 2003) while those at the KR site were positive (1987, 1991, and 1997) (Figure 3, 7). However, it is noteworthy, that despite no CPY in 2003 at KR, all chronologies were characterized by a growth depression beginning in 2003 (Figure 4). Climate correlations mainly differentiated provenance trials, indicating a high diversity of the sites in terms of climatic conditions. However, some climatic drivers seemed to be common for most provenances at both sites (e.g., SPEI3, SPEI6 and PDSI for July for RWI or T of previous April, P and SPEI3; Figure 8 and Supplementary Figure S3A). Namely, RWI chronologies were mainly influenced by drought conditions of summer and autumn months, in particular over the period May – August (Figure 8C,D). However, for the generally drier KR site an additional sensitivity to September – October drought-indices was apparent (Figure 8D and Supplementary Figure S3A). Moreover, T, P, and CWB appeared as climate parameters mainly responsible for the growth of provenances in KR site. In particular, T, P and CWB of previous August and September as well as P and the CWB of May and June, were significantly correlated with RWI (Figure 8A,B and Supplementary Figure S3B). Among the correlations with drought indices (mainly SPEI3 and SPEI6 but also PDSI and CWB), the provenance WT was clearly separated from the other provenances in KN. The SPEI3 and SPEI6 indices, and to a lesser extent PDSI and CWB for the months of the preceding growing season, correlated negatively with RWI. In comparison, climate correlations revealed a more homogeneous growth response of provenances in KR compared to KN, underlining the impression derived from PCGA that between-provenance growth patterns are more similar in KR and more heterogeneous in KN (Figure 8).

### Climate-Shift Effect

**FIGURE 9:**
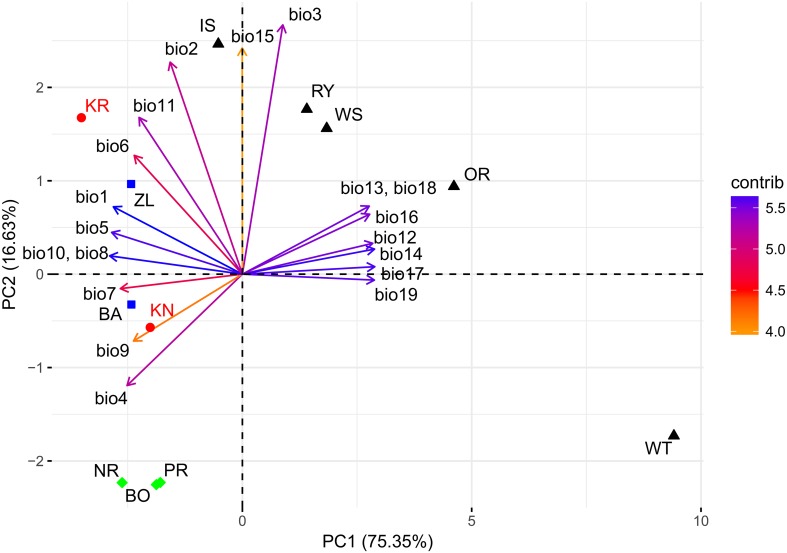
Climate-related variability between provenances and experimental sites based on bioclimatic indexes. Explanations: KR, KN – Kórnik and Knyszyn testing site; BO, PR, NR – northern provenance group, BA, ZL – eastern provenance group, IS, WS, RY, OR, WT – southern provenance group. Variables vector color according to their contribution in total variance: orange – low, blue – high.

The PCA of the 19 bioclimatic variables representing the two trial sites as well as the 10 Norway spruce provenance origins successfully allowed for reducing the multidimensionality to two dimensions. That is, the first two principal components (PC) were able to explain 91.98% of the overall variance (Figure 9 and Table 1). The first PC explained 75.35% of variance and was positively correlated (*r* > 0.25) with precipitation (bio12-14, bio16-19) and negatively correlated (-0.26 < *r* < -0.20) with annual mean temperature and mean temperature of the growing season (bio1, bio4-11). The second component (PC2) explained 16.63% of variance (Table 1) and was positively correlated (0.32 < *r* < 0.51) with the mean monthly temperature range, isothermality, mean temperature of the coldest quarter and precipitation seasonality (bio2, bio3, bio11, and bio15, respectively). Furthermore, PC2 was negatively correlated with temperature seasonality (bio4, *r* < -0.23).

The northern provenances (BO, PR, and NR) formed a cluster, indicating their similarity in terms of their climate origin, while another cluster was formed by the southern provenances (IS, RY, WS and OR) (Figure 9). However, the observed spread of southern provenances was much wider compared to northern provenances. Only the eastern provenances (BA and ZL) and the provenance from the Tatry Mountains (WT) were not included in any cluster. WT was located on the periphery of the PC biplot, which resembles that it originates from the highest location above sea level. It is important to highlight the mutual location of the provenances and the two sites. The distance between the sites and the provenances in relation to the PC axes provides information about the effect of the climatic shift associated with the change of the climate at the trial sites in comparison to the respective provenance origin. For instance, the distance between the scores of the two sites to the provenance WT depicts that it experienced the greatest climatic shift. Moreover, bioclimatic conditions differentiating the origin of the WT provenance to the KN site were mostly related to PC1 while at the KR site they were related to both PC1 and PC2.

## Discussion

Our study confirmed the importance of site marginality for provenance-specific climate sensitivity within provenance trials, showing that optimal climate conditions enhance provenance differentiation (Knyszyn site) while adverse growing conditions resulting from a negative water balance may reduce the effect of provenance-specific growth patterns (Kórnik site). In particular the higher homogeneity of growth patterns between provenances as indicated by the PCGA as well as the more homogeneous climate correlations point toward a weaker differentiation between provenances at KR, i.e., the site with marginal climate conditions. These findings indicate, that provenance-specific growth reactions likely diminish toward the climatic marginality of the considered tree species. This has two important implications in the context of adapting forestry to climate change. First of all, based on our study, site marginality seems to be an important factor to consider when evaluating data from provenance trials. Secondly, even though provenances may express contrasting responses to adverse climate conditions nowadays, their response to anticipated climate conditions may be less diverse. Nonetheless, foresters should aim at selecting provenances which currently cope best with adverse conditions, but it seems noteworthy that even for those provenances, species-specific climatic margins cannot be surpassed. To study the extent to which these provenances can cope with anticipated climate change, marginal sites might be considered candidates of particular interest.

### Individualistic Growth Response and Provenance Clustering

According to the pairwise principal component gradient analysis of provenances, two provenances were characterized by a distinct growth behavior: WT and WS in Kórnik and WT and BA in Knyszyn (Supplementary Figures S1, S2, respectively). The uniform individualistic growth reaction of trees representing the provenance WT indicates relatively low within-population variability and therefore confirms the long-term effect of the selection pressure (Dudley, 2004). Moreover, in case of WT Liebig’s law of the minimum can be applied both at the level of provenance chronologies as well as at the individual tree level (Stine and Huybers, 2017). This suggests that almost all trees of WT responded to a common growth factor and not as in case of other provenances to different local growth factors (Chapin et al., 2011). In turn, WS and BA provenances were only characterized by a homogeneous single-tree reaction under specific environmental conditions. As for WT, this likely relates to their bioclimatic distance to the trial Knyszyn in the case of BA and the bioclimatic distance to the trial Kórnik in the case of WS (Figure 9). This observation may be interpreted as an effect of artificially transferred populations (Eriksson et al., 1980).

The cluster analyses clearly separated the two experimental sites from each other (Figure 5). This confirms results of previous studies that covered a much larger series of provenance trials and which observed a site-specific clustering, too (Karlsson et al., 2001; Suvanto et al., 2016). If we assume that one of the main drivers of Norway spruce growth behavior is water balance – which remarkably differs between Kórnik and Knyszyn – then site-related provenance grouping seems reasonable (Lévesque et al., 2013; Rehschuh et al., 2017). The special position of WT in the cluster analysis likely relates to the special bioclimatic position of this provenance which originates from the highest elevation with the highest annual precipitation sums, hence seems to be determined by the ecological distance between climatic conditions at the location of origin and location of planting, i.e., the Knyszyn site (Figure 9 and Table 1). Surprisingly, in the sub-cluster representing Kórnik the provenance WT formed a small cluster with the southern provenances WS, RY and IS (Figure 5). This however is in line with Koprowski and Zielski (2006) who found that Norway spruce provenances from the so-called “spruceless area” have more similar climate-growth relationships compared to those from southern Poland.

However, another reason for the observed grouping pattern may be the distribution of genetic diversity in Norway spruce related to migration events (Chen et al., 2019). A recent study on Norway spruce pollen distribution in sediments confirmed that the recolonization of Poland by spruce occurred from both the north-west and the south-east (Kupryjanowicz et al., 2018). Moreover, during the last two centuries a massive import of tree seeds in Europe and a shift from natural regeneration to manual seeding significantly affected the genetic structure of Norway spruce populations (Myking et al., 2016).

### Climate-Driven Between-Provenance Variation

Based on the performed climate correlations, drought appeared as the main growth-limiting factor for all provenances (Figure 8 and Supplementary Figure S3). Nevertheless, a site-related variation in the sensitivity to drought stress was indicated by different CPYs in Kórnik compared to Knyszyn (Figure 3). Two negative CPYs in Knyszyn likely relate to severe drought events in 2000 and 2003 widely reported throughout Europe (e.g., Rebetez et al., 2006; He et al., 2018). In contrast, in Kórnik three positive CPYs were recorded in 1987, 1991, and 1997, of which at least one (1997) may have been the result of high precipitation sums in western and southern Poland (Knight and Le Comte, 1998; Kundzewicz et al., 1999). However, based on the inspection of indexed ring-width series (Figure 4) it is obvious that Norway spruce in Kórnik also reacted negatively to the heat wave of 2003. That is, all provenance chronologies in KR showed a growth decline in 2003 which was followed by a growth depression that lasted until 2006. Evidently, the metric on which pointer year analysis is based (Cropper values of a moving window spanning five years) was unable to identify 2003 as a pointer year because the values after 2003 also were comparably low. From this we conclude that the effect of the 2003 heat wave was even more pronounced in Kórnik and eventually resulted in a bark-beetle outbreak which underlines the drought susceptibility and affectedness of trees in Kórnik. Thus, the widely known effect of the cumulative impact of stress load may effectively diminish the genetic variability of provenance-specific growth patterns (Rolland and Lempérière, 2004; Netherer et al., 2015). According to Marini et al. (2012), Norway spruce is more prone to bark beetle outbreaks when growing under climate conditions warmer than those of its historical climatic range.

Among provenances in Knyszyn which did not reduce growth in response to 2003 drought year were WT and WS, both characterized by a long ecological distance to Knyszyn site conditions (Figure 7A, 9). In contrast, provenances BO, PR, and RY did not react to the less severe drought in 2000. Hence the susceptibility of provenances to extreme weather events under moderate climate conditions (as in Knyszyn) appears to be a more complex process, related to adaptation to local conditions in relation to their climate origin – a phenomenon which is known as the transfer effect (Mátyás, 1994).

While exploring between-provenance variation in climate sensitivity the effect of mortality has to be considered since according to Mátyás and Bozic (2009) the genetic tolerance limit of adaptation to adverse climatic conditions may end up in mass mortality. That is, initial effects of climatic extremes such as drought may increase the population’s sensitivity to other abiotic and biotic threats eventually leading to increased rates of mortality (Mátyás et al., 2010). In course of the growth depression observed in KR following the hot and dry summer of 2003 (Figure 4), an increased mortality of Norway spruce was observed, which underlines the importance of mortality at marginal sites. Unfortunately, we lack detailed data to assess whether specific provenances were particularly affected by drought-triggered bark-beetle infestation and eventual die-back. However, it provenance-specific tree vulnerability to bark-beetle infestation seems possible, wherefore future investigations should consider obtaining according information when evaluating data from provenance trials.

Regarding the climate correlations, a detailed analysis of the provenance susceptibility to drought conditions (mainly visible in the SPEI integrated over three and six month periods but also temperature) demonstrates a relatively uniform response of all provenances on the Kórnik site and, in turn, a differentiated reaction on Knyszyn site (Figure 8A,C,D). In the case of Knyszyn site, the distinct climate reaction of the WT provenance is clearly visible, but interestingly, unlike in case of the principal component gradient analysis, is not observable in Kórnik. Thus, a significant effect of provenance and site interaction as obtained with the generalized linear model (Table 4) is supported by the climate correlations, however most likely due to the separate reaction of the WT provenance. In conclusion, it seems that provenances expressed more diverse growth patterns and climate correlations in Knyszyn, while growth patterns and climate correlations were more homogeneous in Kórnik. The higher homogeneity of growth patterns and correlations in Kórnik we interpret as a common reaction to more adverse – in this case dryer – growing conditions.

### Site and Provenance Interaction and Phenotypic Plasticity

Significant effects of the site, provenance and year, and their interactions in the generalized linear model suggest different climate and site-related adaptive responses of the provenances (Table 4). Significant effects of site and provenance interaction may indicate between-provenances variation in plasticity (Dudley, 2004). The site and provenance interaction was mainly found to be significant in studies covering a wide range of environmental conditions (Karlsson et al., 2001; Stojnic et al., 2015; Suvanto et al., 2016), however it generally appears when in one environment genetic variation is almost unnoticeable while it is high in another one (Windig et al., 2004). This is confirmed by our study, where at the site of adverse growing conditions (Kórnik) genetic variation was barely noticeable while under optimal conditions (Knyszyn) between-provenance variation was pronounced. This is consistent with results of Androsiuk and Urbaniak (2014) who stated that the location of a provenance trial influences the level of genetic polymorphism and the patterns of inter-population differentiation.

## Conclusion

Although common garden experiments are considered one of the best tools to simulate the response of trees to anticipated climatic conditions, interpreting the results requires acknowledging the complexity of the phenotypic adaptation process. Besides the intra- and inter-population genetic variation, it is essential to consider the transfer effect expressed in the phenotypic reaction in dependence of the bioclimatic distance between the climate of provenance origin and the climate of the trial site. Moreover, the climatically determined site potential defines the match between actual environmental conditions and ecological needs of the species. That is, the limiting climate conditions of the experimental site may diminish differences of climate-growth responses among provenances which in turn may hamper the appropriate selection of phenotypically stable populations. Consequently, some provenances may better cope with extreme weather under moderate climate conditions, while all provenances homogeneously will respond with growth depression to adverse conditions. In view of the above, it seems reasonable to select sites with moderate climate conditions for provenance trials when aiming at identifying phenotypes which cope best with single extreme events. On the other hand, when seeking to explore the general performance of a species under extreme climate conditions (thus prevailing over several years), sites with more adverse climatic conditions should be considered.

## Data Availability

All datasets generated for this study are included in the manuscript and/or the Supplementary Files.

## Author Contributions

MKl and AB performed ring width analyses and PCGA analyses. JU was in charge of the generalized linear model. MKl, MKo, and RP performed the climatic analyses. MKl and RP performed the bioclimatic analyses. MKl wrote the first draft of the manuscript. All authors gave a substantial contribution to the conception and design of the study, wrote specific sections of the manuscript, and contributed to manuscript revision, read and approved the submitted version.

## Conflict of Interest Statement

The authors declare that the research was conducted in the absence of any commercial or financial relationships that could be construed as a potential conflict of interest.

## Footnotes

1 http://www.noaa.gov/

2 https://climexp.knmi.nl
