# Supplementary material for: Limitations at the Limit? Diminishing of Genetic Effects in Norway Spruce Provenance Trials
Source: Front Plant Sci. 2019 Mar 13;10:306. doi: 10.3389/fpls.2019.00306 (PMC6425888; doi:10.3389/fpls.2019.00306)

Supplementary Material

Limitations at the limit? Diminishing of genetic effects in Norway spruce provenance trials

M. Klisz^1^*, A. Buras^2^, U. Sass-Klaassen^2^, R. Puchałka^3^, M. Koprowski^3^, J. Ukalska^4^

*** Correspondence:** Marcin Klisz, Department of Silviculture and Genetics, Forest Research Institute, Braci Leśnej Street, No 3, Sękocin Stary, 05-090 Raszyn, Poland.

m.klisz@ibles.waw.pl

# Supplementary Figures and Tables

## Supplementary Figures

**Supplementary Figure 1.** PCGA-loadings for detrended ring-width indices for pairwise comparison of Norway spruce provenances in KR site. Red and blue arrows represent X and Y provenance trees, where X is provenance mentioned in the biplot header as the first and Y as the second acronym. *, ** and *** demarcate a significant difference in PCGA-ranks between provenances (p < 0.05, p < 0.01 and p < 0.001, respectively ).

**
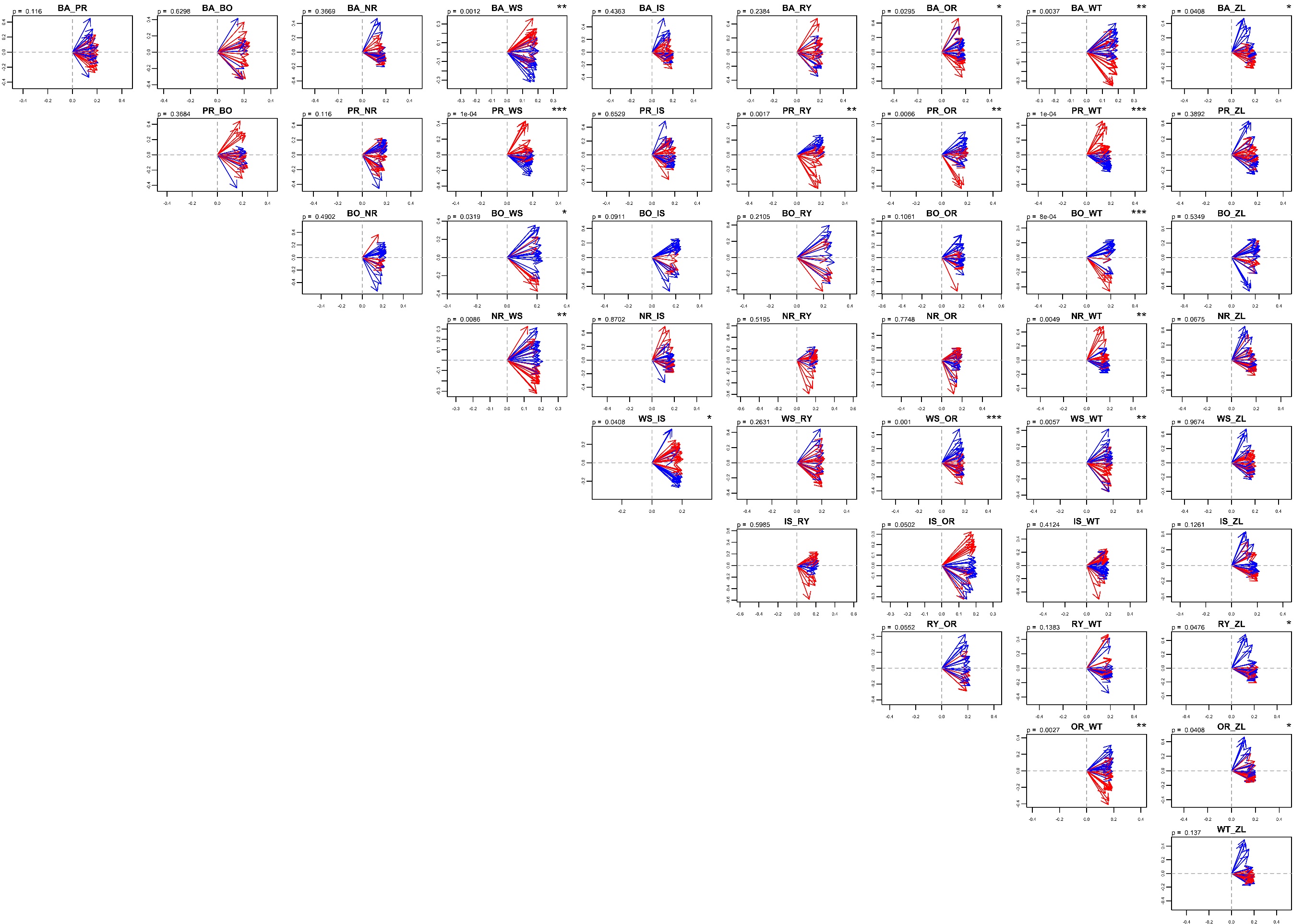
**

**Supplementary Figure 2.** PCGA-loadings for detrended ring-width indices for pairwise comparison of Norway spruce provenances in KN site. Red and blue arrows represent X and Y provenance trees, where X is provenance mentioned in the biplot header as the first and Y as the second acronym. *, ** and *** demarcate a significant difference in PCGA-ranks between provenances (p < 0.05, p < 0.01 and p < 0.001, respectively ).


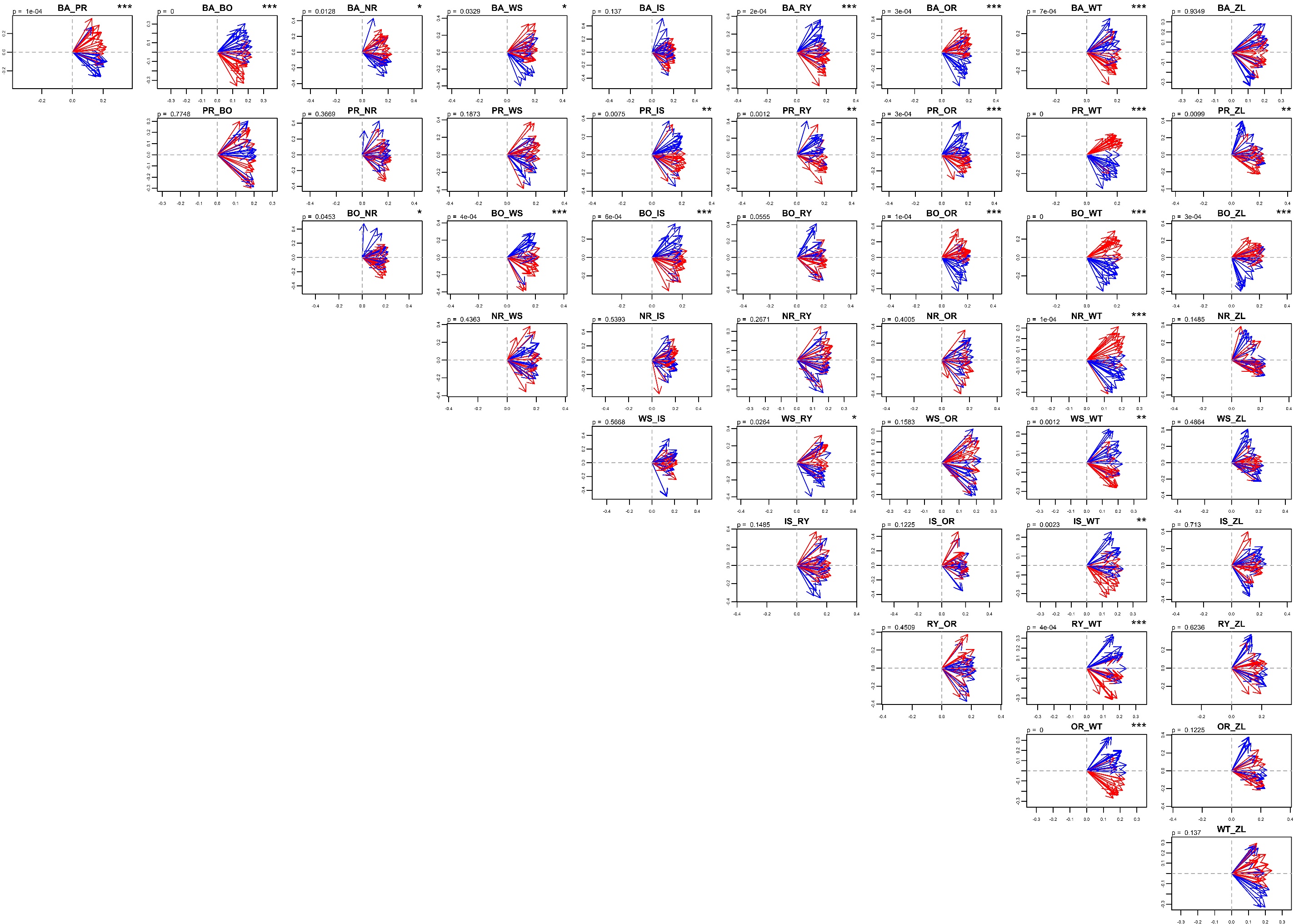


**Supplementary Figure 3.** Correlations of the RWI provenance chronologies with monthly climatic parameters, previous year March through current year October: Palmer drought severity index (panel A), climatic water balance (panel B), over the period 1981–2014 at the two sites: KR, KN. Colors represent correlation coefficient, non-significant correlations are not represented (white), *, ** and *** demarcate a significance level of correlation (p < 0.05, p < 0.01 and p < 0.001, respectively).


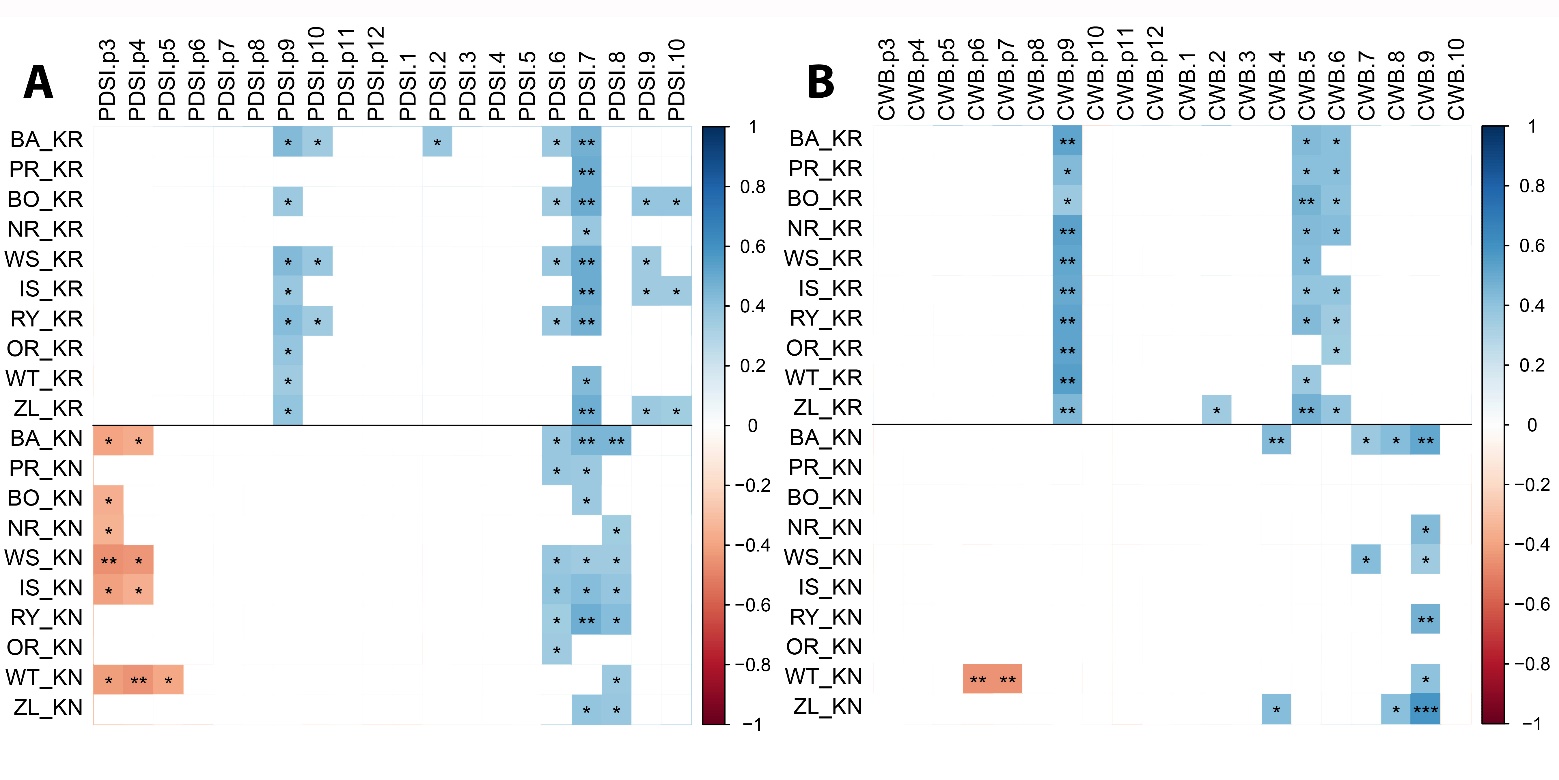

Supplement: Supplementary file 1 [file Data_Sheet_1.docx]
